# Simultaneous glutamine metabolism and PD-L1 inhibition to enhance suppression of triple-negative breast cancer

**DOI:** 10.1186/s12951-022-01424-7

**Published:** 2022-05-06

**Authors:** Yuxia Tang, Siqi Wang, Yang Li, Chen Yuan, Jie Zhang, Ziqing Xu, Yongzhi Hu, Haibin Shi, Shouju Wang

**Affiliations:** 1 Laboratory of Molecular Imaging, Department of Radiology, The First Affiliated Hospital of Nanjing Medical University, Nanjing, Jiangsu China; 2 Department of Interventional Radiology, The First Affiliated Hospital of Nanjing Medical University, Nanjing, Jiangsu China

**Keywords:** MoS_2_ nanosheets, Anti-PDL1, V9302, Glutamine metabolism inhibitor, Triple-negative breast cancer

## Abstract

Blockade of programmed cell death 1 ligand (PD-L1) has been used to treat triple-negative breast cancer (TNBC), and various strategies are under investigation to improve the treatment response rate. Inhibition of glutamine metabolism can reduce the massive consumption of glutamine by tumor cells and meet the demand for glutamine by lymphocytes in tumors, thereby improving the anti-tumor effect on the PD-L1 blockade therapy. Here, molybdenum disulfide (MoS_2_) was employed to simultaneously deliver anti-PDL1 antibody (aPDL1) and V9302 to boost the anti-tumor immune response in TNBC cells. The characterization results show that MoS_2_ has a dispersed lamellar structure with a size of about 181 nm and a size of 232 nm after poly (l-lysine) (PLL) modification, with high stability and biocompatibility. The loading capacity of aPDL1 and V9302 are 3.84% and 24.76%, respectively. V9302 loaded MoS_2_ (MoS_2_-V9302) can effectively kill 4T1 cells and significantly reduce glutamine uptake of tumor cells. It slightly increases CD8^+^ cells in the tumor and promotes CD8^+^ cells from the tumor edge into the tumor core. In vivo studies demonstrate that the combination of aPDL1 and V9302 (MoS_2_-aPDL1-V9302) can strongly inhibit the growth of TNBC 4T1 tumors. Interestingly, after the treatment of MoS_2_-aPDL1-V9302, glutamine levels in tumor interstitial fluid increased. Subsequently, subtypes of cytotoxic T cells (CD8^+^) in the tumors were analyzed according to two markers of T cell activation, CD69, and CD25, and the results reveal a marked increase in the proportion of activated T cells. The levels of cytokines in the corresponding tumor interstitial fluid are also significantly increased. Additionally, during the treatment, the body weights of the mice remain stable, the main indicators of liver and kidney function in the blood do not increase significantly, and there are no obvious lesions in the main organs, indicating low systemic toxicity. In conclusion, our study provides new insights into glutamine metabolism in the tumor microenvironment affects immune checkpoint blockade therapy in TNBC, and highlights the potential clinical implications of combining glutamine metabolism inhibition with immune checkpoint blockade in the treatment of TNBC.

**Graphical Abstract:**

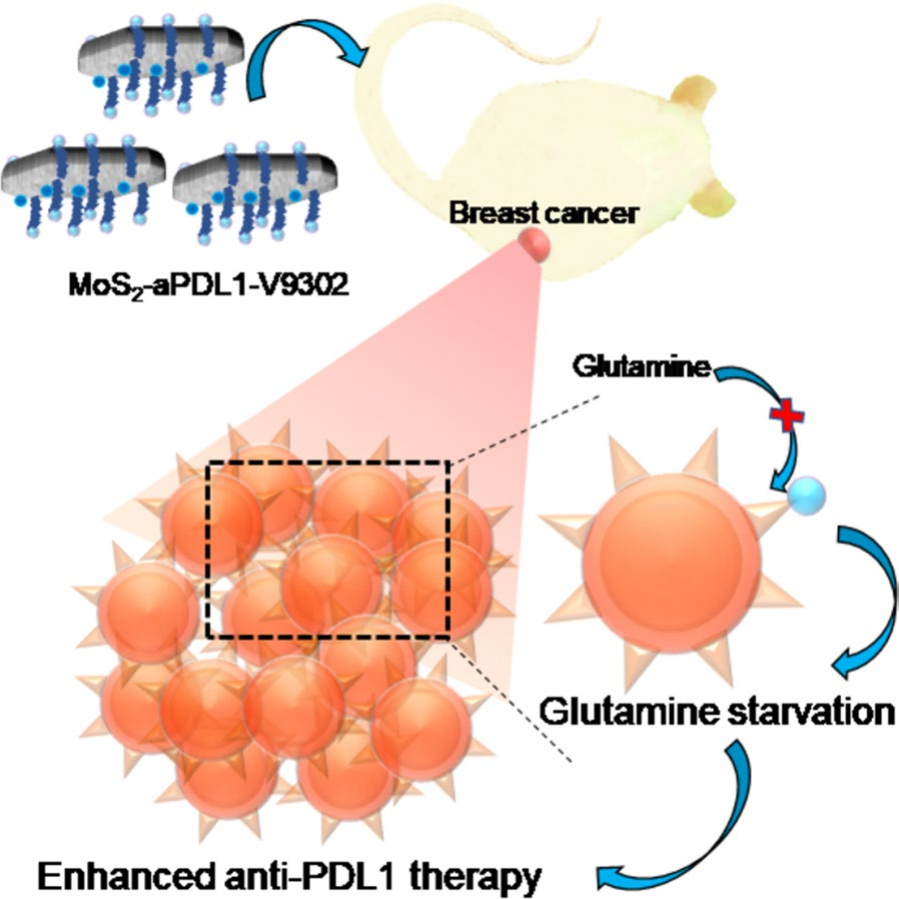

**Supplementary Information:**

The online version contains supplementary material available at 10.1186/s12951-022-01424-7.

## Introduction

Triple-negative breast cancer (TNBC) is difficult to treat since it does not express estrogen receptors, progesterone receptors, and human epidermal growth factor receptor 2 [1, 2]. The development of immunotherapy in recent years has provided new strategies for the treatment of tumors and studies have confirmed that TNBC highly expresses PD-L1, which provides the possibility of anti-PDL1 immunotherapy [3]. However, clinical trials show that the objective response rate of PD-1/PDL1 inhibitors in TNBC patients is only about 20% [4, 5], which is not as good as the chemotherapy drug vinorelbine (25–45%) and cisplatin (32.6%) [6, 7]. In addition, the incidence of serious immune-related adverse reactions in TNBC patients after treatment with PD-1/PDL1 inhibitors is from 5.6% to 9.5%, including death, cytokine release syndrome, neutropenia, fatigue, and peripheral neuropathy [8]. Therefore, exploring new methods to improve the therapeutic effect of PDL1 inhibitors in TNBC and reduce immune-related adverse reactions is an important issue to be solved urgently.

One of the critical factors that restrict the response of immunotherapy is the activation of T cells, which need the energy provided by glutamine [9]. In TNBC, the rapid proliferation of tumor cells consumes a large amount of glutamine, thereby depriving glutamate uptake of lymphocytes and affecting the anti-tumor activity of T cells [10]. When glutamine of tumor cells is restricted, the glutamine uptake of tumor-infiltrating cells increases and enters the tumor core to enhance immune response [11]. Glutamine also plays an important role in the homeostasis and biosynthesis of cells and studies have demonstrated that the inhibition of glutamine can improve the function of CD8^+^ T cell [12]. Various kinds of glutamine inhibitions have been reported to block the glutamine metabolism pathway in cancer cells and enhance the killing effect of T cells [13]. For example, glutamine metabolism inhibitor 6-diazo-5-oxo-l-norleucine can comprehensively antagonize glutamine and has been investigated in a series of clinical trials, but the severe toxicity results in the abandonment of them [14]. Recently, an effective glutamine transporter inhibitor V9302 has been reported to selectively targets alanine-serine-cysteine transporter 2 and inhibit glutamine uptake [15].

It is worth noting that V9302 has also been found to be able to change the location of tumor-infiltrating lymphocytes from the periphery of the tumor to the core of the tumor [16]. It is well-known that the distribution of tumor-infiltrating lymphocytes is a critical factor that restricts the response of anti-PDL1 therapy in TNBC [17]. Therefore, it can be speculated that the combination of V9302 and anti-PDL1 antibody appears to robustly activate the proliferation of lymphocytes and improve their function, and also enhance the efficacy of anti-PDL1 therapy. However, like other small molecule drugs, V9302 is poor water solubility, rapidly cleared in the body, poor tissue permeability, and systemic toxicity, limiting its application. Therefore, it is of great significance to develop suitable carriers for drug delivery.

Designing a nano-delivery system can not only protect the loaded drug from rapid degradation during the systemic circulation but also increase the distribution of the drug at the tumor site and provide a platform for targeted drug delivery [18, 19]. The two-dimensional nano-material molybdenum disulfide has a large specific surface area, a high surface free energy, and an ultra-thin structure, making it easier to penetrate biological membranes, and has attracted great attention in the field of drug delivery and tumor immunotherapy [20].

The delivery of glutamine metabolism inhibitor V9302 through molybdenum disulfide (MoS_2_) can specifically change the location of CD8^+^ T cells in the core of TNBC, which can effectively relieve immunosuppression, ultimately enhancing the therapeutic effect of anti-PDL1 therapy on TNBC. In addition, V9302 is an effective glutamine metabolism inhibitor, which can enhance the function of lymphocytes and reverse the nutritional deprivation of immune cells by tumor cells. This work aims to investigate the co-delivery of immune activation of glutamine metabolism inhibitor and anti-PD-L1 by MoS_2_ nanocarriers and the therapeutic efficacy in TNBC.

## Results and discussion

### Preparation and characterization of MoS_2_-aPDL1-V9302

**Fig. 1:**
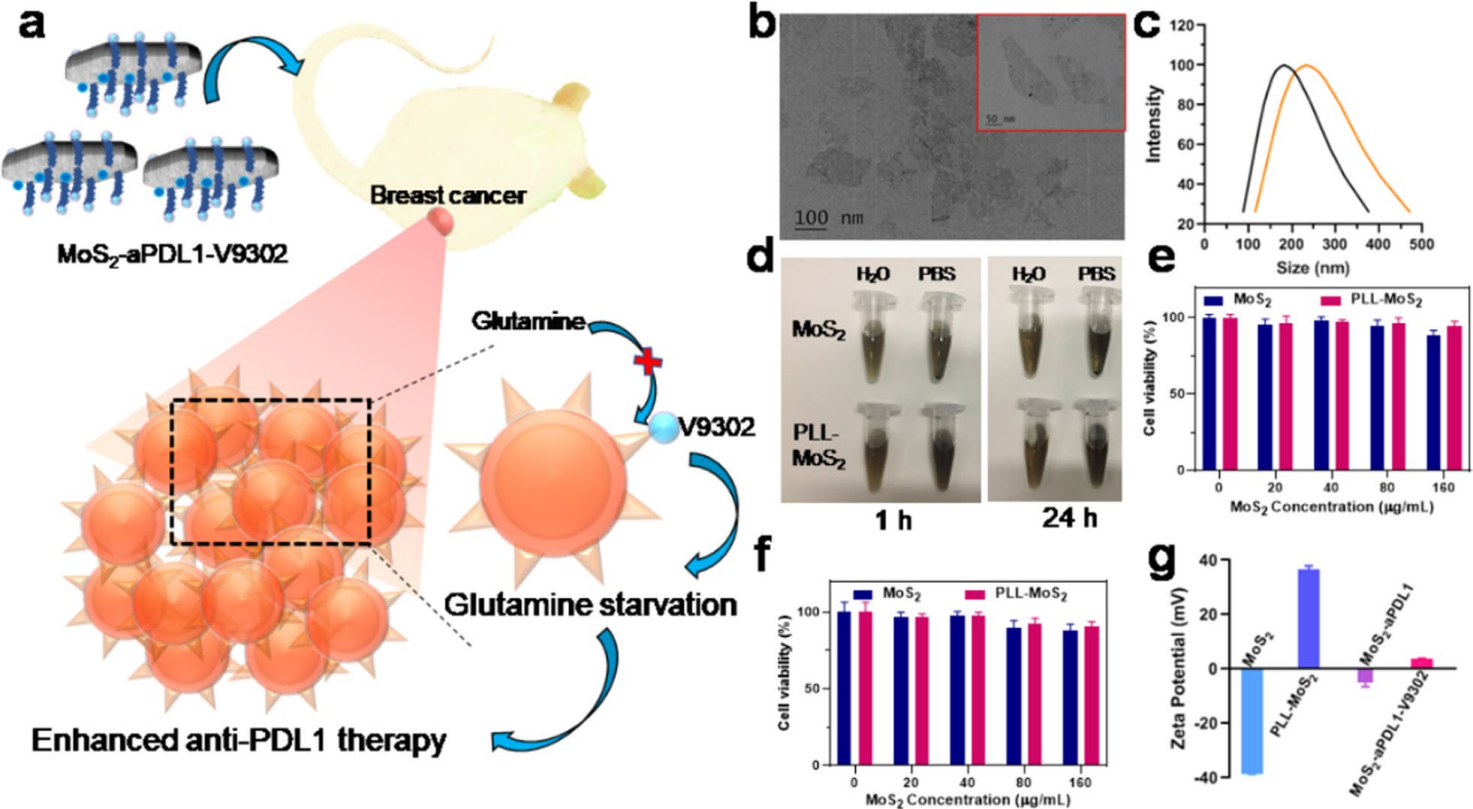
Characterization of MoS_2_. **a** Schematic of simultaneous PD-L1 and glutamine metabolism inhibition to enhance suppression of triple-negative breast cancer. V9302 targets the glutamine transporter on the surface of breast cancer cells to inhibit the transport of glutamine and result in glutamine starvation of tumor cells. Then the glutamine levels in tumor interstitial fluid increase and more CD8^+^ cells enter the tumor core, thereby improving anti-PDL1 efficacy and synergistically inhibiting tumor growth. **b** Morphology of MoS_2_ observed by TEM. Inset: TEM image with high magnification. **c** Hydrodynamic diameters of MoS_2_ before and after the modification of PLL by DLS analysis. **d** The photograph of MoS_2 was_ dispersed in water and PBS for 1 h and 24 h. The cell viability of 4T1 cells treated with MoS_2_ and MoS_2_-PLL for **e** 24 h and **f** 48 h. **g** Zeta potentials of MoS_2_, PLL-MoS_2_, MoS_2_-aPDL1 and MoS_2_-aPDL1-V9302. Data are presented as means ± SD (n = 5)

Glutamine is the most abundant non-essential amino acid in the human body, and it is one of the energy sources of cancer cells [22]. The high expression of glutamine transporter in breast cancer is significantly correlated with the number of PDL1 and PD1-positive and FOXP3-positive cells [23]. The glutamine metabolism in triple-negative breast cancer depends on its correlation with immune cell types to varying degrees, so changes in glutamine metabolism can affect the infiltration and activation of immune cells [24]. To enhance the treatment of TNBC, V9302 is used to inhibit the glutamine uptake of cancer cells and increase the glutamine level in tumor interstitial fluid, which can improve the efficacy of anti-PDL1 therapy (Fig. 1a). Transmission electron microscopy (TEM) images show that MoS_2_ are monodisperse and irregularly flakes (Fig. 1b). The dispersion of MoS_2_ in PBS shows obvious precipitation after standing for 1 h and 24 h, but it remains stable after PLL modification, indicating that the dispersibility is improved (Fig. 1c). Dynamic light scattering (DLS) reveals that MoS_2_ and PLL-MoS_2_ have a size of 181 nm and 232 nm, respectively (Fig. 1d). Then MTT experiments were used to evaluate the cytotoxicity of MoS_2_ and PLL-MoS_2_ on 4T1 cells. When incubated at a concentration of up to 160 µg/mL for 24 h, about 89% and 95% of cells survive in MoS_2_ and PLL-MoS_2_ groups, respectively (Fig. 1e). With the incubation time further prolonged to 48 h, the cell viability decreases, but 90% of cells are still alive in the PLL-MoS_2_ group (Fig. 1f). These results confirm that PLL-MoS_2_ has no obvious toxicity at concentrations up to 160 µg/mL, suggesting excellent biological compatibility. Zeta potential of MoS_2_, PLL-MoS_2_, MoS_2_-aPDL1 and MoS_2_-aPDL1-V9302 are − 38 mV, 37 mV, 5 mV, and 4 mV, respectively (Fig. 1g). The change of zeta potential demonstrates the successful loading of aPDL1 and V9302. Quantitative analysis shows that PLL-MoS_2_ has a loading capacity of 3.84% and 24.76% for aPDL1 and V9302, respectively. Drug release tests show that 86.8% of V9302 was released from MoS_2_-V9302 in PBS at pH 5.5 for 120 h. In contrast, in PBS with pH 7.4, 13.8% of V9302 was released (Additional file 1: Fig. S1).

### Effect of MoS_2_-V9302 on cell viability, glutamine metabolism, and CD8^+^ cells distribution

**Fig. 2:**
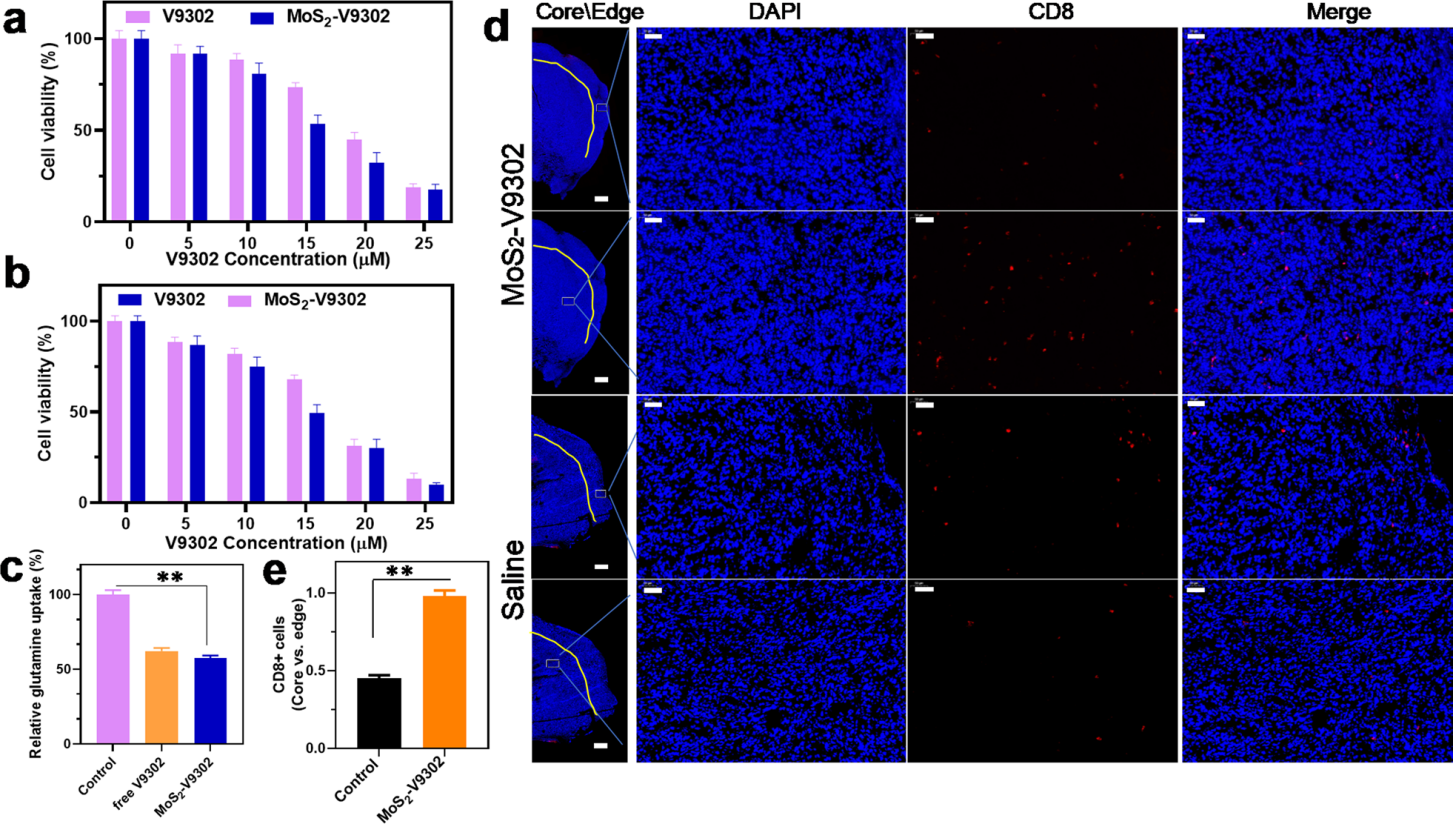
V9302/MoS_2_-V9302 inhibits glutamine metabolism and improves CD8^+^ cells in the tumor core. Cytotoxicity of MoS_2_-V9302 in 4T1 cells incubated for **a** 48 h and **b** 72 h. **c** The glutamine uptake of 4T1 tumor cells treated by V9302/MoS_2_-V9302. **d** Immunohistochemistry of CD8 (red) in tumor sections. Nuclei were stained with DAPI (blue). The tumor was divided into Edge (< 500 μm from tumor margin) and core (> 500 μm) regions by a yellow solid line. Scale bars: 50 μm. **e** CD8^+^ cells were counted and calculated from 3 fields of view. **P < 0.01

Due to the poor water solubility of free V9302, it usually needs to be dissolved in the toxic organic solvent dimethyl sulfoxide [25]. To avoid the use of organic solvents, MoS_2_ was employed to deliver it to tumor cells. To investigate the effect of MoS_2_-V9302 on 4T1 breast cancer cells, MTT assays were performed to evaluate the viability of cells treated with free V9302 and MoS_2_-V9302. The results show that when incubated for 48 h and 72 h, V9302 and MoS_2_-V9302 have concentration-dependent cytotoxicity (Fig. 2a and b). For each concentration, MoS_2_-V9302 has a similar or slightly better effect than the corresponding free V9302, confirming that the use of MoS_2_ delivery does not hinder the drug's effect, and can overcome the disadvantage of poor water solubility of V9302 for further application. It has been previously reported that the uptake of glutamine in TNBC is increased to maintain cell proliferation and function, the inhibition of glutamine uptake can interfere with the growth of TNBC [13, 26]. We also observed that when incubated with MoS_2_-V9302, the glutamine uptake of 4T1 breast cancer cells significantly decrease than that of the control group, and slightly lower than that of the free V9302 group (Fig. 2c) and a significant increase in both glucose uptake and lactate production than those of control group (Additional file 1: Fig. S2). Compared with the free drug, the inhibition rate of glutamine metabolism after drug loading was slightly higher. Then the CD8^+^ T cells were detected by using flow cytometry and the CD8^+^ cells are slightly increased after the treatment of MoS_2_-V9302 compared with saline (Additional file 1: Fig. S3). The distribution of CD8^+^ T cell was investigated by histological assessment and the results show that after the treatment of MoS_2_-V9302, the infiltration of CD8^+^ T cells to the tumor core (> 500 μm from the tumor margin) are increased, in comparison; the restriction of CD8^+^ T cells to the tumor periphery is observed in the control (Fig. 2d). Given that the change of localization of CD8^+^ T cells from the periphery to the deeper tumor site can increase anti-PDL1 therapy response, it is a potential strategy to combine V9302 and anti-PDL1 therapy in TNBC.

### In vivo distribution and therapeutic efficacy

**Fig. 3:**
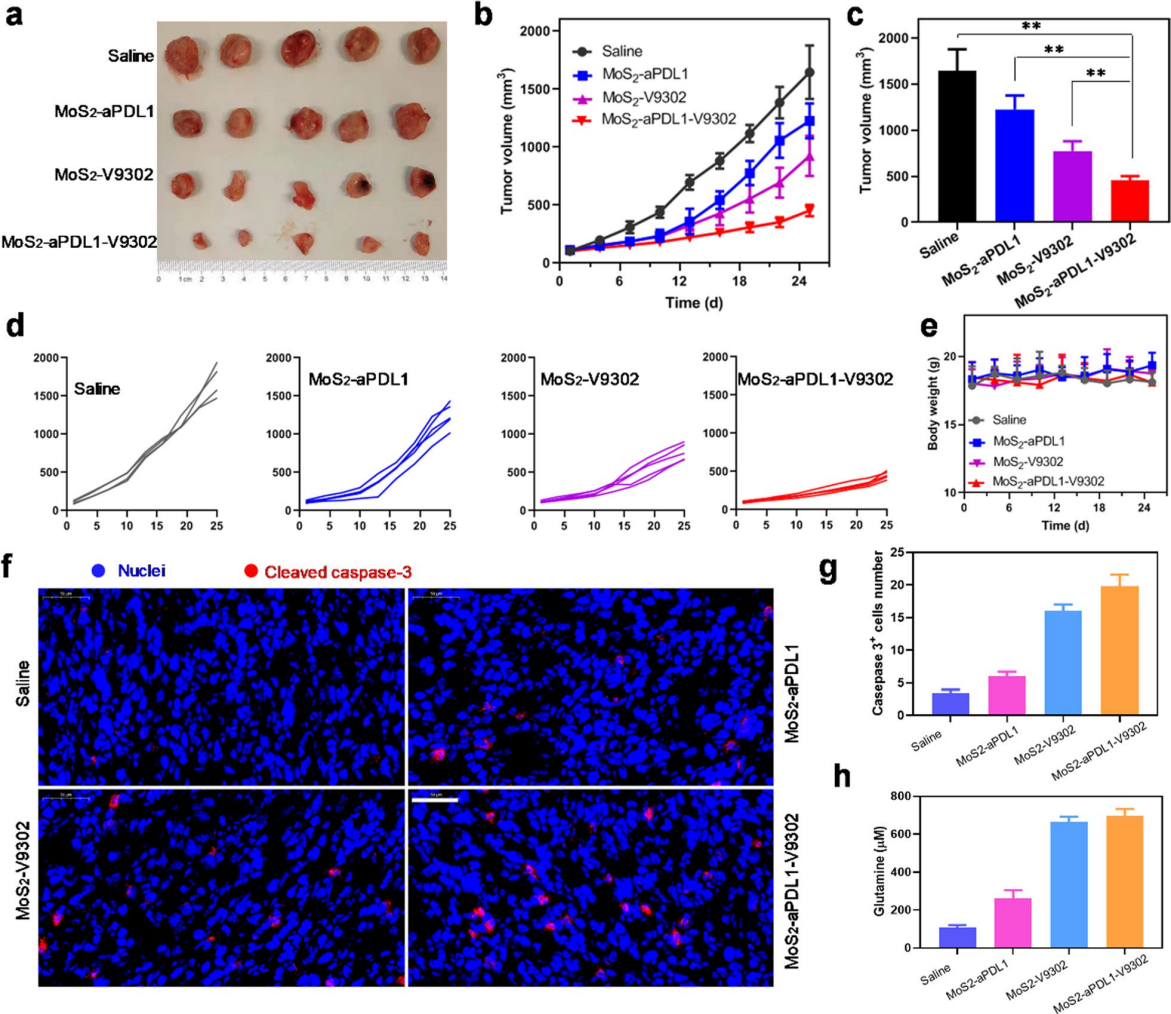
Tumor inhibition of MoS_2_-aPDL1-V9302 in 4T1 tumor model. **a** Photo of the tumors dissected from 4T1 tumor-bearing mice 25 days after the first treatment. **b** Tumor growth curves in different groups. **c** Tumor volume at the end of the treatment. **P < 0.01. **d** The tumor growth curve in each group. **e** Body mass of mice during the treatment. **f** Immunofluorescence of cleaved caspase-3 in tumor sections. Blue: Nuclei stained with DAPI. Red: cleaved caspase-3. Scale bars: 50 μm. **g** The number of cleaved caspase-3 positive cells was counted from 3 fields of view. **h** The concentration of glutamine in tumor interstitial fluid

The accumulation of the MoS2/PLL nanosheets in tumors was assessed in a mouse breast cancer model by intravenously injecting MoS2/PLL-Cy3, and the tumors of the mice showed obvious fluorescence at 24 h postinjection (Additional file 1: Fig. S4), suggesting that MoS2/PLL can accumulate in tumor for further drug delivery. MoS_2_-V9302 has been confirmed to efficaciously induce 4T1 cell death, the effect was further investigated in vivo and the combination efficacy of V9302 and anti-PDL1 was also evaluated. Orthotopic 4T1 tumors were treated with different nano drugs daily for 5 days and the tumor volumes were monitored every three days. As shown in Fig. 3a, the tumors are smaller in the MoS_2_-aPDL1-V9302 group. The tumor growth curves show the same trend (Fig. 3b). The mice injected with saline were set as control and the tumor volumes increased rapidly. The monotherapy of MoS_2_-aPDL1 and MoS_2_-V9302 exhibits modest antitumor efficacy on 4T1 tumors. The combination therapy achieved by MoS_2_-aPDL1-V9302 leads to an obvious boost of antitumor activity. Statistical results showed that the improvement of the efficacy of the combined treatment group compared with the control group and the single drug treatment group was statistically significant (Fig. 3c). Individual tumor growth curves more clearly reveal the growth of tumor volume in each group over time, indicating that the combination therapy group has the best effect (Fig. 3d). The efficacy of free aPDL1 + V9302 was investigated and the tumor growth curves are shown in Additional file 1: Fig. S5. The relative tumor volume of free aPDL1 + V9302 and MoS_2_-aPDL1-V9302 is 55.56% and 36.95%, respectively. These results demonstrate that MoS_2_-aPDL1-V9302 shows a better treatment effect. The monitoring of the body mass of the mice throughout the process shows that the weight of the mice remains stable without significant loss, indicating a high degree of tolerance for this treatment modality (Fig. 3e). Cleaved caspase-3 was stained on the 4T1 tumor section and more positive cells are observed in MoS_2_-V9302 and MoS_2_-aPDL1-V9302 groups (Fig. 3f). The corresponding quantitative results show a more significant (threefold) increase in cleaved caspase-3 positive cells (Fig. 3g). MoS_2_-aPDL1 has only a marginal effect on glutamine concentrations within the interstitial fluid of 4T1 tumors. Notably, the glutamine concentrations of MoS_2_-V9302 and MoS_2_-aPDL1-V9302 groups are 664 and 696 μM, which are significantly higher than saline and MoS_2_-V9302 groups (Fig. 3h).

### Immunoresponse assessments

**Fig. 4:**
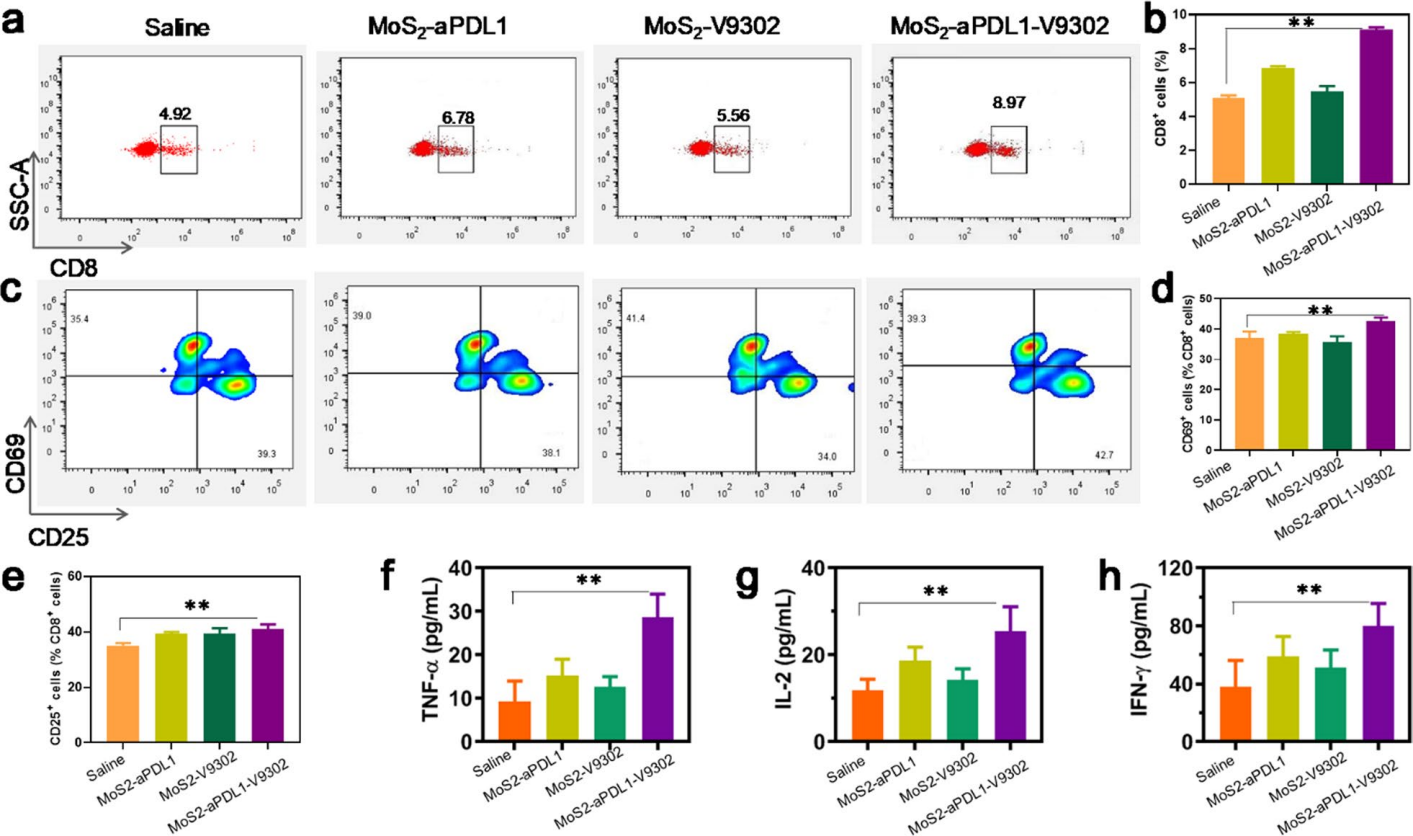
MoS_2_-aPDL1-V9302 increases the ratio of cytotoxic T lymphocytes in tumors and the cytokine levels in tumor interstitial fluid. **a** The ratio of cytotoxic T lymphocytes (CD8^+^) was detected by flow cytometry in tumors after different treatments. **b** The corresponding quantitation of CD8^+^ cells. **c** The ration of CD69^+^ and CD25^+^ cells in CD8^+^ cells and the corresponding quantitation of **d** CD69^+^ and **e** CD25^+^ cells. The levels of various cytokines include **b** TNF-α, **c** IL-2, and **d** IFN-γ in tumor interstitial fluid. Each group has 5 samples. **P < 0.01

The 4T1 tumors treated with various drugs were collected and the CD8^+^ cytotoxic T lymphocytes were quantified by flow cytometry. The tumors treated with MoS_2_-aPDL1-V9302 show a 1.78-fold increase in CD8^+^ T cells in tumor single-cell suspension compared with the saline group (Fig. 4a and b). The number of CD8^+^ cells also increased to varying degrees in MoS_2_-aPDL1 and MoS_2_-V9302, slightly lower than in the combination treatment group. The cell surface glycoprotein CD69, an important marker of T cell activation, is expressed at very low basal levels in resting lymphocytes, and once activated, its expression increases significantly and CD69 is one of the earliest markers upregulated after T cell activation [27]. CD25 plays a key role in responsiveness to IL-2, enabling T lymphocyte activation and further IL-2 secretion [28]. Herein, the percentage of CD69^+^ and CD25^+^ in CD8^+^ cells was further detected. As Fig. 4c–e show, in comparison with saline, MoS_2_-aPDL1-V9302 demonstrated a marked increase in CD69^+^ and CD25^+^ cells. Both MoS_2_-aPDL1 and MoS_2_-V9302 resulted in a slight increase of CD69^+^ and CD25^+^ cells.

In addition to directed apoptosis, CD8^+^ T cells can also indirectly kill target cells by releasing cytokines such as IFN-γ, a cytokine capable of inhibiting viral replication and enhancing the presentation of specific antigens [29]. Due to that these cytokines are critical indicators of cellular immunity activation [30], the cytokine levels including TNF-α, IL-2, and IFN-γ in tumor interstitial fluid were measured by the Enzyme-linked immunosorbent assay (ELISA). An overall increase in TNF-α, IL-2, and IFN-γ was observed in tumor interstitial fluid after the treatment with MoS_2_-aPDL1-V9302 (Fig. 4f–g). Together, these results demonstrate that the combination therapy of aPDL1 and V-9302 induce tumor cell death while simultaneously augmenting CD8^+^ cytotoxic T lymphocytes and cytokines within the 4T1 tumor microenvironment.

### Systemic toxicity assessments

**Fig. 5:**
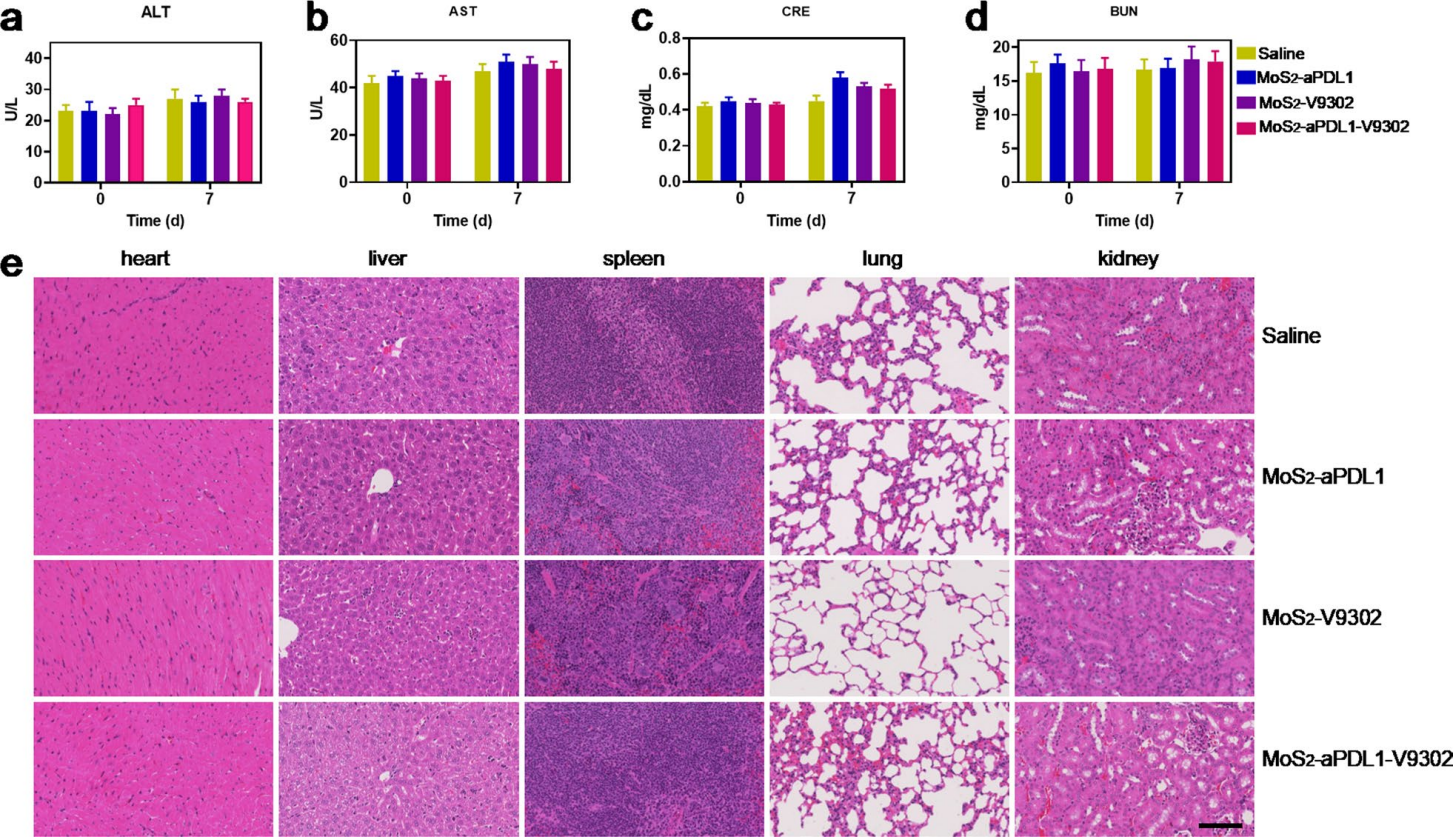
Systemic toxicity assessments. **a** ALT, **b** AST, **c** creatinine, and **d** BUN values of the mice before and after the treatment. **e** Repressive images of hematoxylin–eosin staining of heart, liver, spleen, lung, and kidney. In each group, there are 5 mice

Considering that safety is the top priority for treatment, the systemic toxicity effects of MoS_2_-aPDL1-V9302 were assessed. Hepatic enzymes, including ALT and AST, kidney function index, creatinine, and BUN, were detected and the levels are within the normal region (Fig. 5a–d). Furthermore, there were minor-to-no lesions in the heart, liver, spleen, lung, and kidney of the tumor sections stained by hematoxylin–eosin at 7 days.

## Conclusions

In conclusion, we demonstrate that co-delivery of a glutamine inhibitor V9302 and an immune checkpoint inhibitor aPDL1 with molybdenum disulfide blocks glutamine’s massive uptake by TNBC cells increases glutamine levels in tumor interstitial fluid. It can promote the entry of CD8^+^ cells from the tumor edge into the core, increase the proportion of activated CD8^+^ cells in the tumor, and then improve the therapeutic effect on tumor cells. The further combination of molybdenum disulfide-conjugated PD-L1 antibody and glutamine inhibitor provides a new strategy for the treatment of TNBC.

## Methods

### Materials

MoS_2_ nanosheets and poly(l-lysine) (PLL) were purchased from Sigma-Aldrich (Shanghai, China). V9302 hydrochloride was purchased from MedChem Express (purity = 98.88%, Shanghai China). Anti-mouse PD-L1 (B7-H1) was purchased from Bio X cell (West Lebanon, USA). Dulbecco’s Modified Eagle Medium (DMEM), Fetal Bovine Serum (FBS), and Phosphate Buffered Saline (PBS) were from Thermo Scientific (Waltham, MA). 3-(4,5-dimethylthiazol-2-yl)-2,5-diphenyltetrazolium bromide (MTT), were purchased from Sigma-Aldrich (St. Louis, MO, USA). Glutamine Detection Assay Kit was obtained from Abcam (ab197011). Deionized water (Millipore) with a resistivity of 18 MΩ/cm was used in all experiments.

### Preparation of MoS_2_-aPDL1

MoS_2_-aPDL1 was synthesized according to the previously published paper. Briefly, 0.5 mg/mL (0.5 mL) of MoS_2_ in water was sonicated for 1 h and 0.5 mL of 1 mg/mL poly (l-lysine) (PLL) was added under stirring for 1 h. Then the mixture was incubated overnight at 4 °C followed by concentration two times at 8000 rpm for 30 min to obtain PLL-MoS_2_. PLL-MoS_2_ was resuspended in PBS and mixed with anti-PDL1 antibody (mass ration = 25:1) and the production was purified at 8000 rpm for 20 min at 4 °C. The supernatant was collected, and the concentration of protein was measured at 595 nm by using Bicinchoninic Acid Assay.

### Preparation of MoS_2_-aPDL1-V9302

MoS_2_-aPDL1 solution (containing 1 mg/mL of aPDL1, 0.5 mL) was mixed with V9302 (4 mg/mL, 1.0 mL) and were stirred overnight at 4 °C. Then the mixture was centrifugated (8000 rpm, 20 min) and washed three times. The supernatant was collected and the concentration of V9302 was measured by UV at 276 nm. To obtain MoS_2_-V9302, PLL-MoS_2_ was resuspended in PBS and mixed with V9302 (mass ration = 3:1) and the production was purified at 8000 rpm for 20 min at 4 °C. The supernatant was collected, and the concentration of protein was measured at 276 nm. Then, 5 mg of MoS_2_-V9302 was added to 1 mL PBS with pH 5.5 or 7.4 and shaken in the dark. At different time points, this solution was centrifuged at 8000 rpm for 10 min to collect the supernatant and replaced with 1 mL fresh PBS with a corresponding pH value. The released V9302 was calculated according to the absorbance at 276 nm.

### Cell viability measurement and glutamine metabolism analysis

Cell viability was tested by MTT assay in 4T1 cells. The cells were seeded in 96-well plates at a density of 10^4^ cells/well 24 h before treatment. The medium was replaced by 100 µL of FBS-supplemented medium containing various concentrations of free V9302 or MoS_2_-V9302 and incubated for 48 h. Then the solutions were removed and 100 µL serum-free media containing 10 µL of MTT (5 mg/mL) was added. After 4 h incubation, the absorbance at 490 nm was detected and the relative cell viability (%) was calculated as 100% × A_sample_/A _control_. Each sample had five repeated wells.

For glutamine metabolism analysis, the cells were first cultured in a glutamine-deprived medium for 6 h and then free V9302 or MoS_2_-V9302 in a normal DMEM medium was added cultured for a further 24 h. After trypsin digestion, cells were washed with cold PBS three times. The cells in PBS were sonicated on ice for 1 min and centrifugated at 13,000 rpm for 15 min at 4 °C. The glutamine metabolism and the protein concentrations were detected according to the protocol of the EnzyChrom Glutamine Assay kit and Bicinchoninic Acid Assay, respectively.

The glucose metabolism was evaluated by detecting the concentration of glucose and lactate in the medium of 4T1 cells. After being cultured for 24 h, cells were incubated with MoS_2_-V9302 (the concentration of V9302 was 15 μM) for 48 h with different treatments. Then the medium was collected and measured by Glucose and Lactate Colorimetric/Fluorometric Assay Kit. Cells without any treatment were set as control and each group had three repeat samples.

### CD8^+^ cells distribution analysis

Briefly, orthotopic tumor-bearing mice (Balb/c, female, 6 weeks) were obtained by injecting 4T1 cells (5 × 10^5^) into the number 4 mammary fat pads. After 11 days, the mice were treated with MoS_2_-V9302 (daily, 75 mg/kg) for 5 days, the tumors were harvested, formalin-fixed, and immunohistochemically stained for CD8 and nuclei by Servicebio (Wuhan, China). The distribution of CD8^+^ cells was analyzed by Qupath software. Edge and core were considered < 500 μm and > 500 μm from tumor margin, respectively. The number of CD8^+^ cells was quantified by Image J software. The experiment was repeated three times.

### In vivo biodistribution of MoS2

Cy3-Poly (l-lysine) modified MoS2 nanosheets in 200 μL of PBS were intravenously injected (20 mg/kg) into tumor-bearing Balb/c mice. The mice were sacrificed 24 h post-injection. Tumors and major organs, including heart, liver, spleen, lung, and kidney were harvested for fluorescence imaging by using an IVIS Lumina XR system under a Cy3 filter. The fluorescence intensity of each organ compared with tumor-adjacent muscle tissues was detected using an automated segmentation method.

### In vivo anti-tumor investigation

Orthotopic tumor-bearing mice were obtained using the protocol mentioned above and when the tumor volume reached 100 mm^3^, the mice were divided into four groups (n = 5). The mice were treated with MoS_2_-aPDL1, MoS_2_-V9302, or MoS_2_-aPDL1-V9302 daily for 5 days, and the dose of aPDL1 and V9302 was 10 mg/kg and 75 mg/kg, respectively. The mice injected with saline were set as control. Then the body mass and the short and long diameters were measured every three days. After 4 weeks, the tumors were collected and the interstitial fluid was obtained [21]. Briefly, after being weighed, washed with PBS, and wiped with filter paper, tumors were centrifuged at 100*g* for 30 min at 4 °C in a microcentrifuge tube containing pluriStrainer (20 μm). The interstitial fluid was detected using the Glutamine Detection Assay Kit. The tumor was formalin-fixed and immunohistochemically stained for cleaved caspase 3 and nuclei by Servicebio (Wuhan, China). The number of cleaved caspase 3 cells was analyzed by Image J software in three different high magnification fields of view.

### Immunoresponse and systemic toxicity assessments

Orthotopic tumor-bearing mice were treated with MoS_2_-aPDL1, MoS_2_-V9302, or MoS_2_-aPDL1-V9302 daily for 5 days. Then the tumors were collected and lymphocytes in tumors of the same weight were isolated using the tumor dissociation kit (Miltenyi Biotec, Germany). Then the lymphocytes were incubated with anti-CD8, CD25, and CD69 antibodies and detected by flow cytometer. Cytokine levels including TNF-α, IFN-γ, and IL-2 in tumor interstitial fluid were measured according to the manufacturer’s protocols of the Enzyme-linked immunosorbent assay (ELISA).

To investigate the systemic toxicity, healthy Balb/c mice (female, 8 weeks) were treated with MoS_2_-aPDL1, MoS_2_-V9302, or MoS_2_-aPDL1-V9302 daily for 5 days, and the dose of aPDL1 and V9302 was 10 mg/kg and 75 mg/kg, respectively. At 7 days, the major organs, including the heart, liver, spleen, lung, and kidney, were harvested for hematoxylin–eosin staining. The blood before the treatment and 7 days after the treatment was collected for biochemical analysis, including Alanine aminotransferase (ALT) and aspartate aminotransferase (AST), creatinine (CRE), and urea nitrogen (BUN).

### Statistics

All statistical analyses were performed using R Language. Student’s t-tests, and one- or two-way analysis of variance (ANOVA) were used for the comparisons between 2 groups and multiple comparisons, respectively. P < 0.05 was considered a significant difference.

## Supplementary Information

**Additional file 1: Figure S1.** V9302 release profile from MoS_2_-V9302 incubated in PBS buffer at pH 7.4 and pH 5.5. **Figure S2**. (a) Glucose uptake and (b) lactate production in 4T1 cells incubated with MoS2-V9302 for 24 h. Cells without any treatments were set as control. Data are presented as means ± SD (n = 3) and P values were generated by t-test. **P < 0.01. **Figure S3**. Detection of CD8^+^ T cell infiltration using flow cytometry in 4T1 tumors after the treatment of (a) saline and (b) MoS_2_-V9302. (c) The corresponding quantification results. **Figure S4**. (a) The organs and tumors fluorescence image at 24 h after intravenous injection of the MoS2/PLL-Cy3. (b) Quantified fluorescence intensity of different organs at 24 h after intravenous injection of MoS2/PLL-Cy3. Values reported are the means ± SD, n = 3. **Figure S5**. **(a)** Photo of the tumors dissected from 4T1 tumor-bearing mice 25 days after the first treatment. **(b)** Tumor growth curves in different groups.

## Data Availability

All data used to generate these results is available in the main text.
