# Supplementary material for: Simultaneous glutamine metabolism and PD-L1 inhibition to enhance suppression of triple-negative breast cancer
Source: J Nanobiotechnology. 2022 May 6;20:216. doi: 10.1186/s12951-022-01424-7 (PMC9074360; doi:10.1186/s12951-022-01424-7)
Supplement: Supplementary file 1 — Additional file 1: Figure S1. V9302 release profile from MoS2-V9302 incubated in PBS buffer at pH 7.4 and pH 5.5. Figure S2. (a) Glucose uptake and (b) lactate production in 4T1 cells incubated with MoS2-V9302 for 24 h. Cells without any treatments were set as control. Data are presented as means ± SD (n = 3) and P values were generated by t-test. **P < 0.01. Figure S3. Detection of CD8+ T cell infiltration using flow cytometry in 4T1 tumors after the treatment of (a) saline and (b) MoS2-V9302. (c) The corresponding quantification results. Figure S4. (a) The organs and tumors fluorescence image at 24 h after intravenous injection of the MoS2/PLL-Cy3. (b) Quantified fluorescence intensity of different organs at 24 h after intravenous injection of MoS2/PLL-Cy3. Values reported are the means ± SD, n = 3. Figure S5. (a) Photo of the tumors dissected from 4T1 tumor-bearing mice 25 days after the first treatment. (b) Tumor growth curves in different groups. [file 12951_2022_1424_MOESM1_ESM.docx]

Supporting Information

Simultaneous glutamine metabolism and PD-L1 inhibition to enhance suppression of triple-negative breast cancer

Yuxia Tang ^a^, Siqi Wang ^a^, Yang Li ^a^, Chen Yuan ^a^, Jie Zhang ^a^, Ziqing Xu ^a^, Yongzhi Hu ^a^, Haibin Shi ^b, *^, Shouju Wang ^a, **^

^a^ Laboratory of Molecular Imaging, Department of Radiology, The First Affiliated Hospital of Nanjing Medical University, Nanjing, Jiangsu, China

^b^ Department of Interventional Radiology, The First Affiliated Hospital of Nanjing Medical University, Nanjing, Jiangsu, China.

*Corresponding authors. Email addresses: shihb@vip.sina.com (H. Shi), shouju.wang@gmail.com (S. Wang)

** Corresponding author at Laboratory of Molecular Imaging, Department of Radiology, The First Affiliated Hospital of Nanjing Medical University, Nanjing, Jiangsu, China.


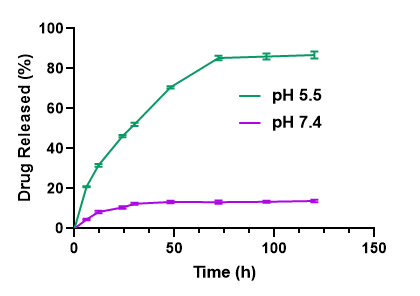


**Figure S1**. V9302 release profile from MoS_2_ -V9302 incubated in PBS buffer at pH 7.4 and pH 5.5.


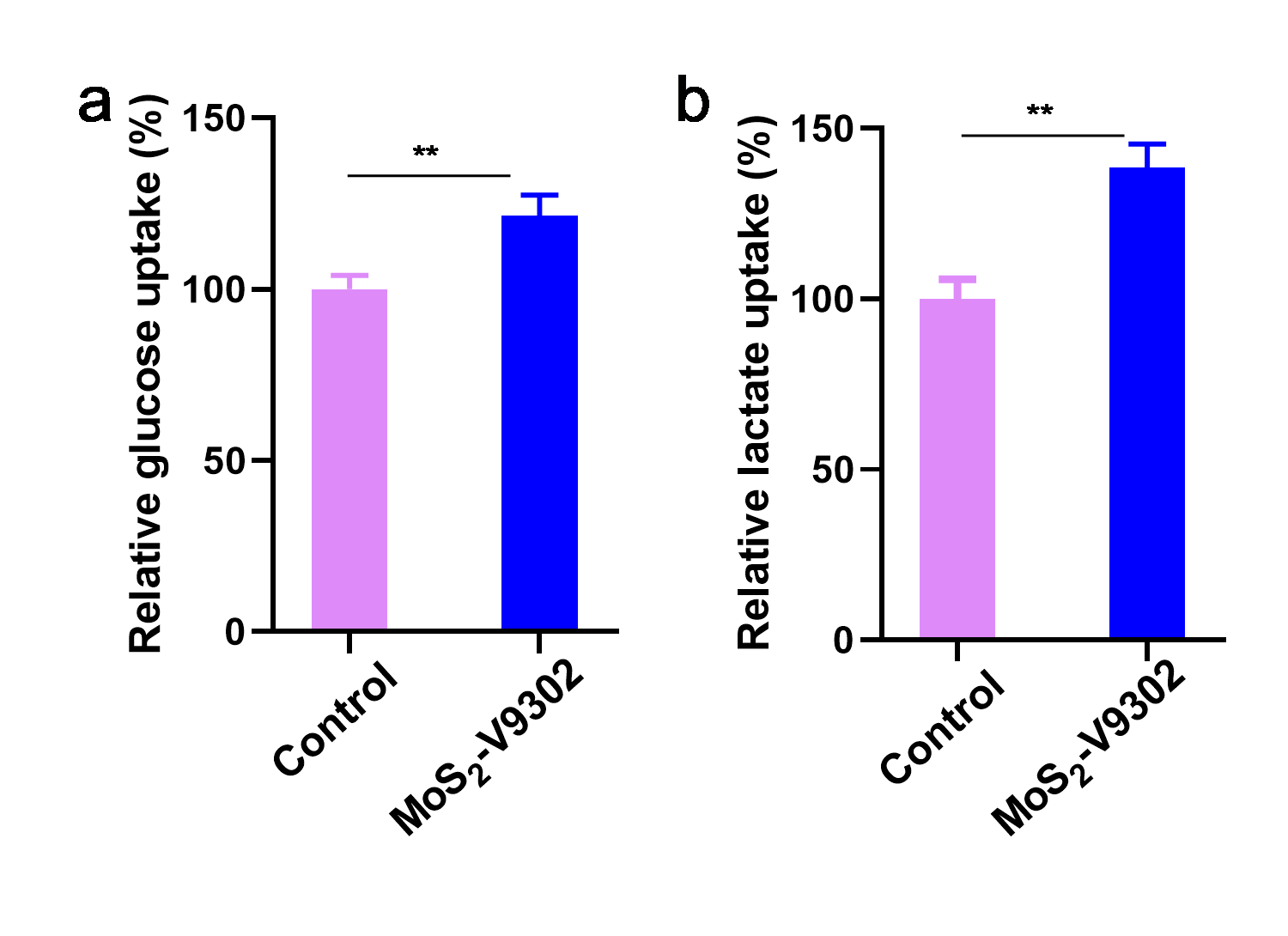


**Figure S2**. (a) Glucose uptake and (b) lactate production in 4T1 cells incubated with MoS2-V9302 for 24 h. Cells without any treatments were set as control. Data are presented as means ± SD (n = 3) and P values were generated by t-test. **P < 0.01.


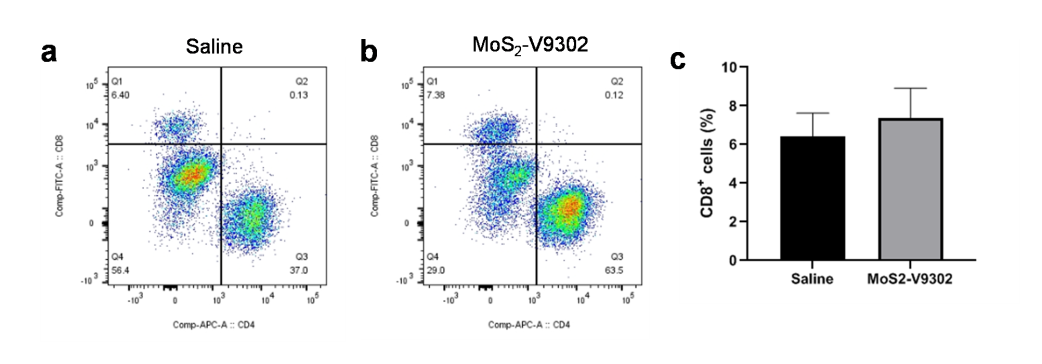


**Figure S3**. Detection of CD8^+^ T cell infiltration using flow cytometry in 4T1 tumors after the treatment of (a) saline and (b) MoS_2_-V9302. (c) The corresponding quantification results.


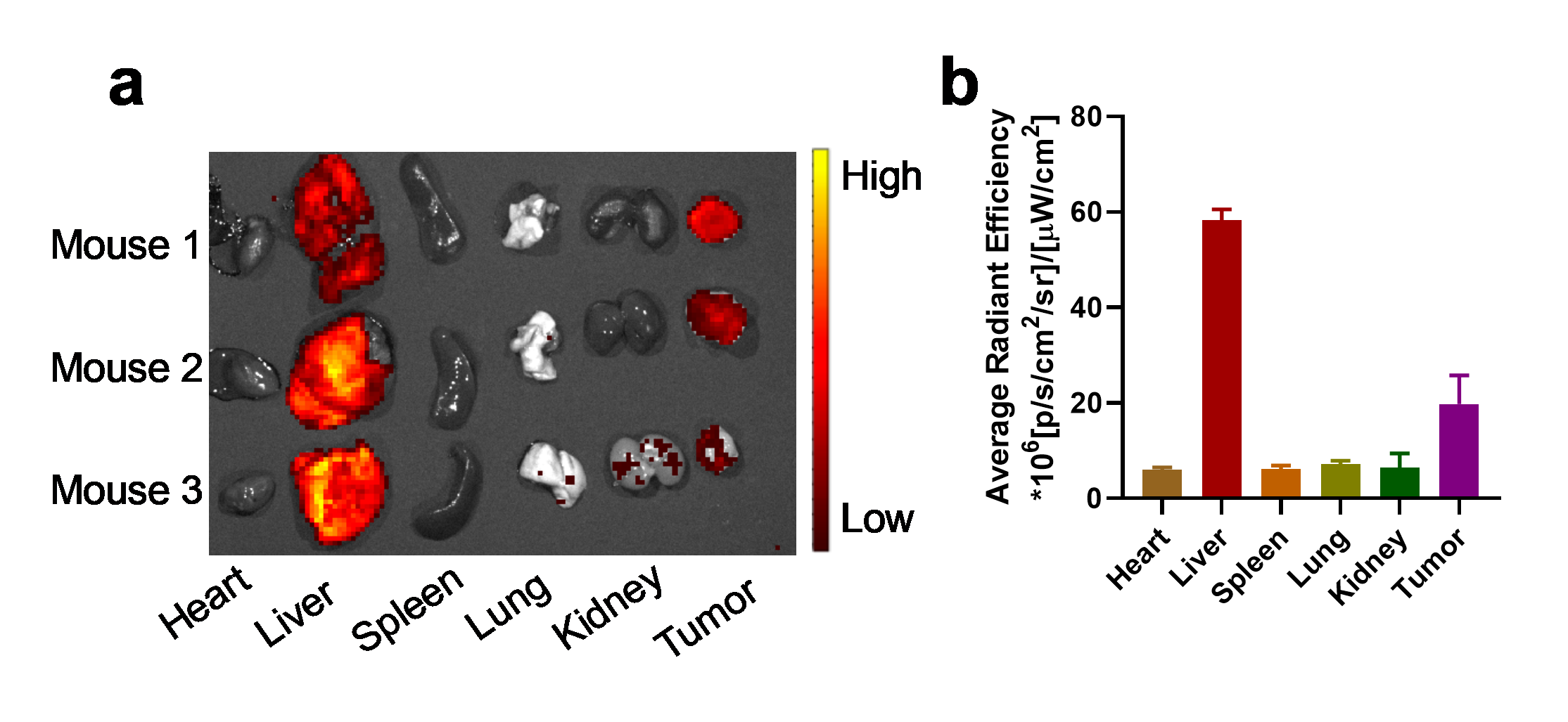


**Figure S4**. (a) The organs and tumors fluorescence image at 24 h after intravenous injection of the MoS2/PLL-Cy3. (b) Quantified fluorescence intensity of different organs at 24h after intravenous injection of MoS2/PLL-Cy3. Values reported are the means±SD, n = 3.


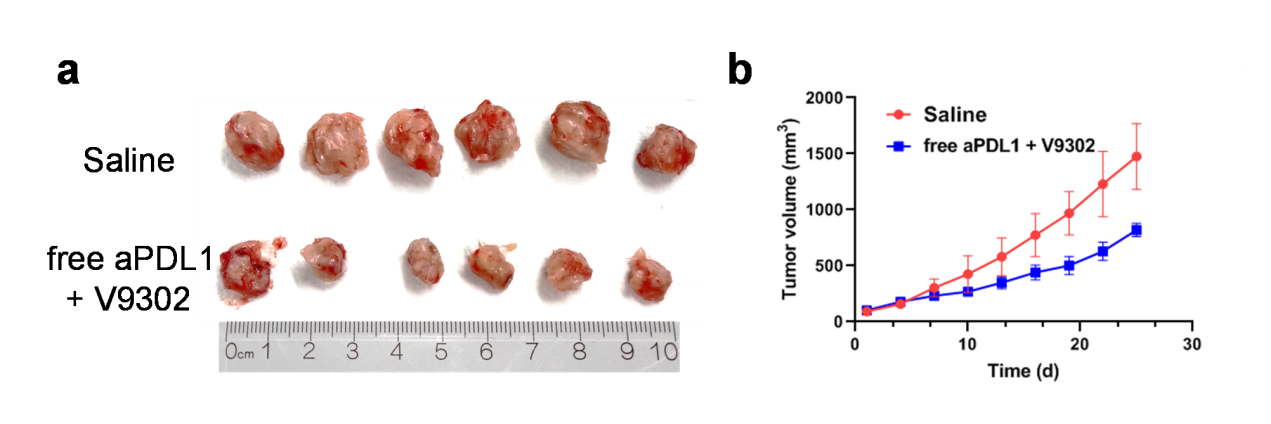


**Figure S5**. **(a)** Photo of the tumors dissected from 4T1 tumor-bearing mice 25 days after the first treatment. **(b)** Tumor growth curves in different groups.
